# Comparison of Journal Self-Citation Rates between Some Chinese and Non-Chinese International Journals

**DOI:** 10.1371/journal.pone.0049001

**Published:** 2012-11-16

**Authors:** Zu-Guo Yang, Feng Gao, Chun-Ting Zhang

**Affiliations:** 1 Library, Tianjin University, Tianjin, China; 2 Department of Physics, Tianjin University, Tianjin, China; University of Nebraska Medical Center, United States of America

## Abstract

**Background:**

The past 3 decades have witnessed a boost in science development in China; in parallel, more and more Chinese scientific journals are indexed by the Journal Citation Reports issued by Thomson Reuters (SCI). Evaluation of the performance of these Chinese SCI journals is necessary and helpful to improve their quality. This study aimed to evaluate these journals by calculating various journal self-citation rates, which are important parameters influencing a journal impact factor.

**Methodology/Principal Findings:**

We defined three journal self-citation rates, and studied these rates for 99 Chinese scientific journals, almost exhausting all Chinese SCI journals currently available. Likewise, we selected 99 non-Chinese international (abbreviated as ‘world’) journals, with each being in the same JCR subject category and having similar impact factors as their Chinese counterparts. Generally, Chinese journals tended to be higher in all the three self-citation rates than world journal counterparts. Particularly, a few Chinese scientific journals had much higher self-citation rates.

**Conclusions/Significance:**

Our results show that generally Chinese scientific journals have higher self-citation rates than those of world journals. Consequently, Chinese scientific journals tend to have lower visibility and are more isolated in the relevant fields. Considering the fact that sciences are rapidly developing in China and so are Chinese scientific journals, we expect that the differences of journal self-citation rates between Chinese and world scientific journals will gradually disappear in the future. Some suggestions to solve the problems are presented.

## Introduction

According to the Journal Citation Reports (JCR), the journal Impact Factor (IF) is calculated by dividing the number of citations in the JCR year by the total number of articles published in the two previous years [1]. In other words, the impact factor is average citations per published item [2]. Introduced by Eugene Garfield and regularly published in the annual updates of the JCR, the impact factor is a fundamental citation-based measure for significance and performance of scientific journals [3].

Journal self-citations, an important subject in scientometrics studies [4], are classified into the self-citing rate and the self-cited rate. It is believed that the self-citing rate relates a journal self-citation to the total number of references it gives, whereas the self-cited rate relates a journal self-citations to the number of times it is cited by all journals, including itself [4], [5]. Self-citation of a journal may affect its impact factor [6], [7].

Historically, many scientists were devoted to studying the issue of journal self-citations [4]–[23]. Most of these studies mentioned in the above references can be classified into four categories.

**Figure 1:**
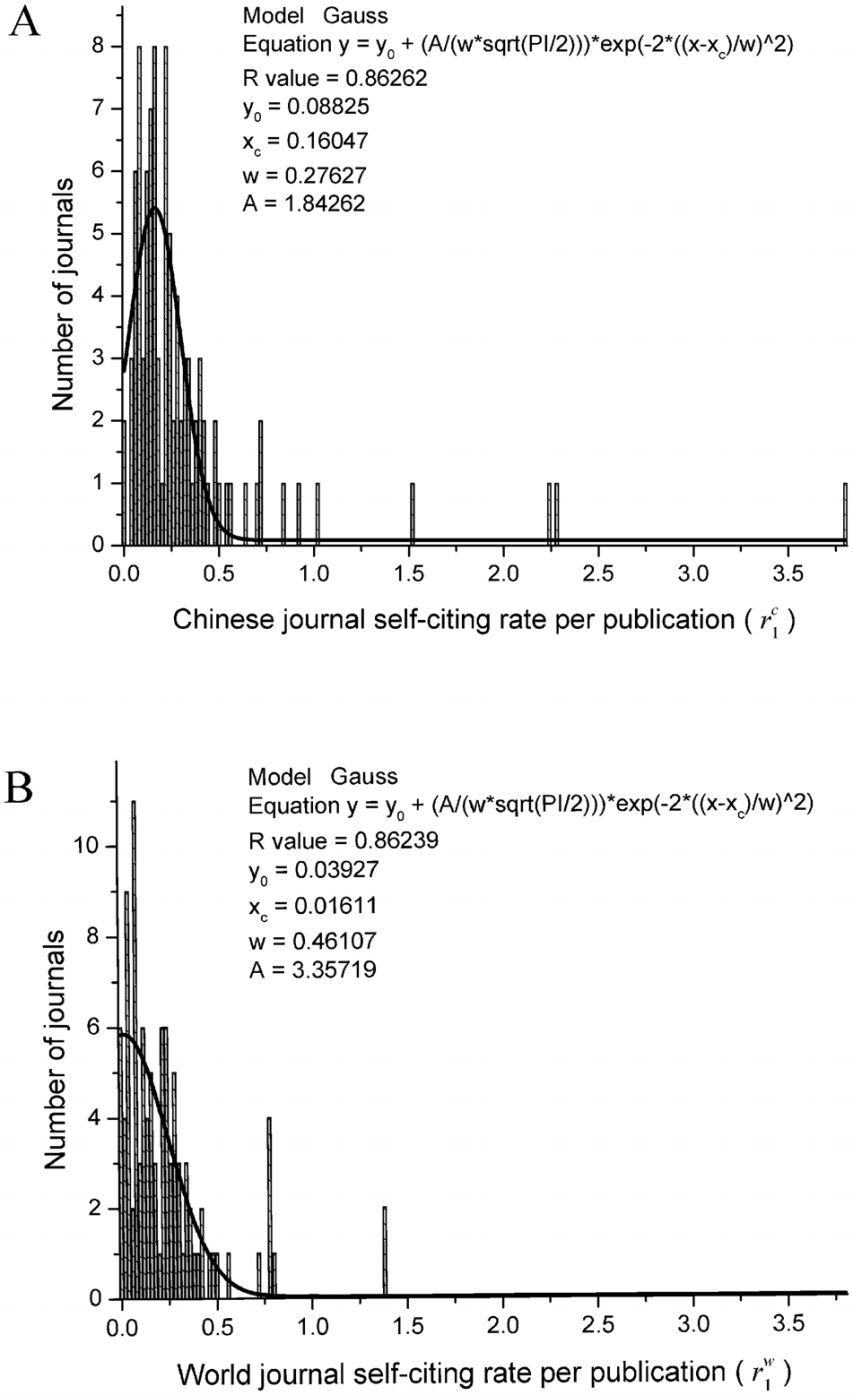
Distributions of the journal self-citing rates per publication (*r_1_*). A) Journal self-citing rates for Chinese journals and B) for world journals. Note that both distributions can be well fitted by a Gaussian model. However, the maximum of the former is at 0.16, whereas that of the latter is at 0.016, indicating that more Chinese journals have greater 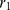 values than their world counterparts. Also note that there is no world journal whose 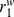 value is greater than 1.5 (refer to B), whereas there are four Chinese journals whose 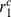 is greater than 1.5. One Chinese journal even has the journal self-citing rate per publication 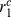 value larger than 3.5 (refer to A).

- The first kind of studies explore the basic characteristics of journal self-citation. Rousseau pointed out that the self-citing and self-cited rates are aspects of the citation structure of journals and found that self-cited rates reach an earlier peak than external-citation [4]. This finding probably represents a basic characteristic of the self-citation of journals. Based on the self-citation data of the most productive semiconductor journals, Tsay found that there is a significant correlation between self-citing rates and self-cited rates of journals [8]. The finding was then confirmed by Biglu, who also found that there is a linear correlation between journal self-citing and self-cited rates [9]. Perhaps, this is another basic characteristic of the self-citation of journals.
- The second kind of studies are devoted to studying the self-citing and self-cited rates of journals of an individual country. For example, Zhang and Yamazaki evaluated 128 Japanese journals indexed by the JCR in terms of impact factors, self-citing and self-cited rates [10]. They found that only 15 Japanese journals, with a wide variation of self-citing and self-cited rates, have obtained a current impact factor higher than 1. Ugolini and Casilli evaluated the visibility of 73 Italian journals indexed by JCR in terms of self-citing and self-cited rates of journals and impact factor etc. [11]. Liu and Wang studied the self-cited rates of 884 Chinese biomedical journals in the year 2005–2007 [12]. They found that the self-citation rates of these Chinese journals had a downward trend rather than an increase trend in the year 2005–2007. Xia and Wu investigated the self-citation rates of 222 Chinese journals in the year 2006–2008 [13]. They found that the average values of self-citation rates of these journals showed a downward trend without significant difference.
- The third kind of studies include the investigations for a certain discipline. For example, Maczelka and Zsindely investigated the dependence of the impact factors and the journal self-citation rates of 22 chemistry journals on the journal age [14]. Tsay investigated the self-citing and self-cited rates of the most productive semiconductor journals [8]. Krauss calculated self-citation rates of 107 journals ranked in the JCR in the subject category “Ecology” and found six journals suspected to request for additional citations showed high self-citation rates [15]. Other investigates include those for anaesthesia journals [6], [16], otolaryngology journals [7] and orthopaedic journals [17].
- The fourth kind of studies are devoted to investigating the journal self-citation rates and the manipulation of their impact factors. The mathematical expressions of the relation between journal self-citation rate and its impact factor were established by Yu and co-workers, and were used to study the issue of manipulation of impact factor [18], [19].

Although Liu and Wang studied the self-cited rates of 884 Chinese biomedical journals [12], and Xia and Wu investigated the self-citation rates of 222 Chinese journals [13], all or most of these journals were not indexed by the JCR. This situation promoted us to begin this study. Here we studied the self-citation rates of 99 Chinese scientific journals indexed by the JCR, which almost exhausted all Chinese journals indexed by the JCR at the time when the present study was performed. For comparison, we performed a similar study for 99 world (international, non-Chinese) journals indexed by the JCR. Detailed comparisons of self-citations between these two kinds of journals were performed.

**Figure 2:**
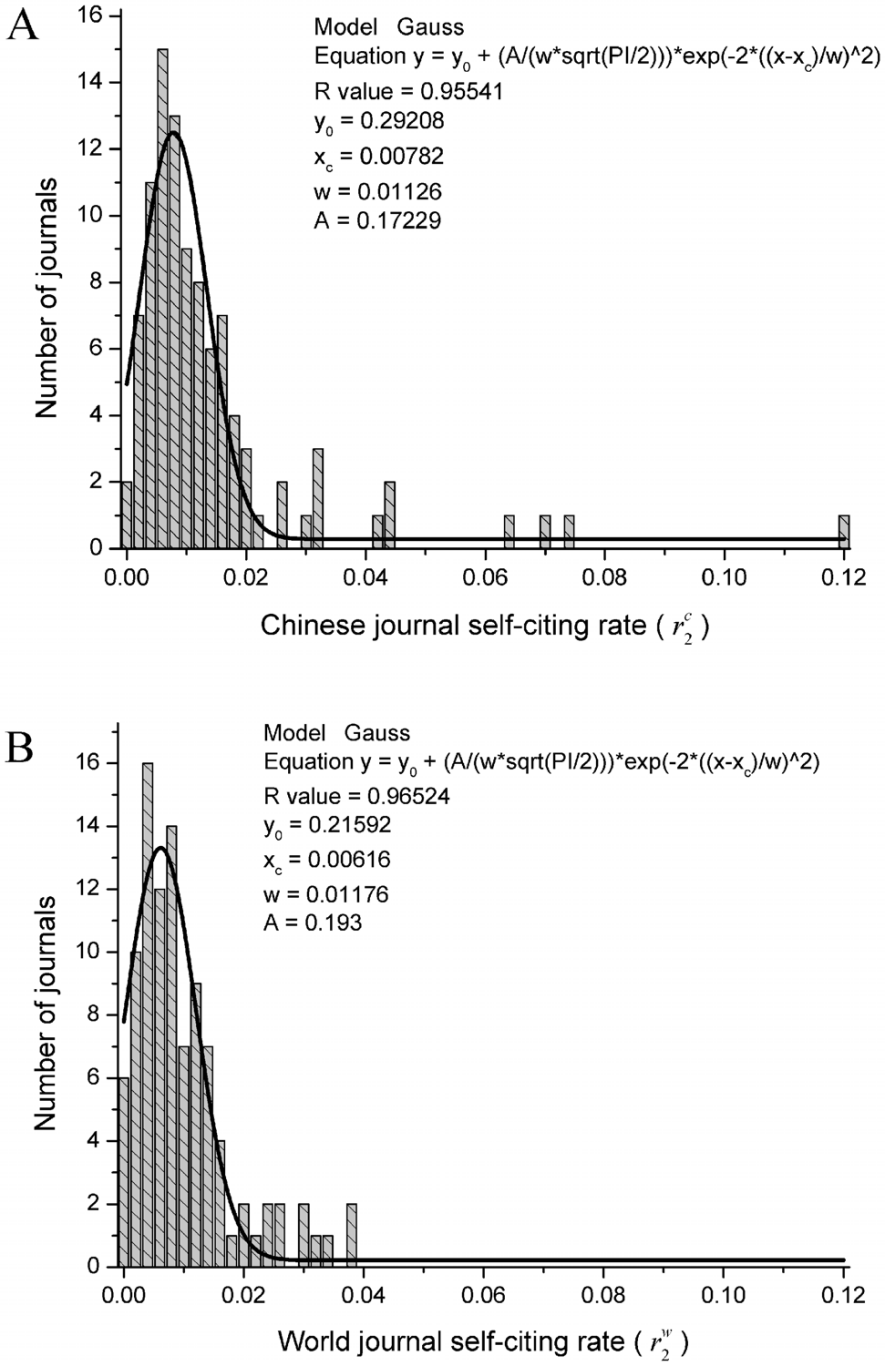
Distributions of the journal self-citing rates (*r_2_*). A) Journal self-citing rates for Chinese journals and B) for world journals. Similar to Fig. 1 A and B, note that both distributions shown can also be well fitted by a Gaussian model. However, the maximum of the former is at 0.008, slightly greater than that of the latter (0.006). Additionally, there is no world journal whose 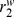 value is greater than 0.04, whereas there are seven Chinese journals whose 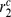 values are greater than 0.04. One Chinese journal even has the journal self-citing rate 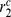 larger than 0.119. Both facts indicate that the Chinese journal self-citing rates are generally higher than those of world journals.

## Results and Discussion

### Comparison of the Distributions of Journal Self-citation Rates between Chinese and World Journals

Fig. 1 shows the distributions of journal self-citing rates per publication for both Chinese and world journals. As we can see from Fig. 1 A and B, both distributions of journal self-citing rates per publication of Chinese and world journals can be well fitted by a truncated Gaussian (shortly as Gaussian) model. However, the maximum of the former is slightly greater than that of the latter. Fig. 1 A and B show that the maximum of the distribution for Chinese journals is at about 0.16, whereas that for world journals is approximately equal to 0.016. Additionally, there is no world journal whose 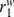 value is greater than 1.5, whereas there are four Chinese journals whose 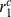 is greater than 1.5. One Chinese journal even has the journal self-citing rate 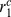 value larger than 3.5. Both facts indicate that the Chinese journal self-citing rates per publication were generally higher than those of world journals.

**Figure 3:**
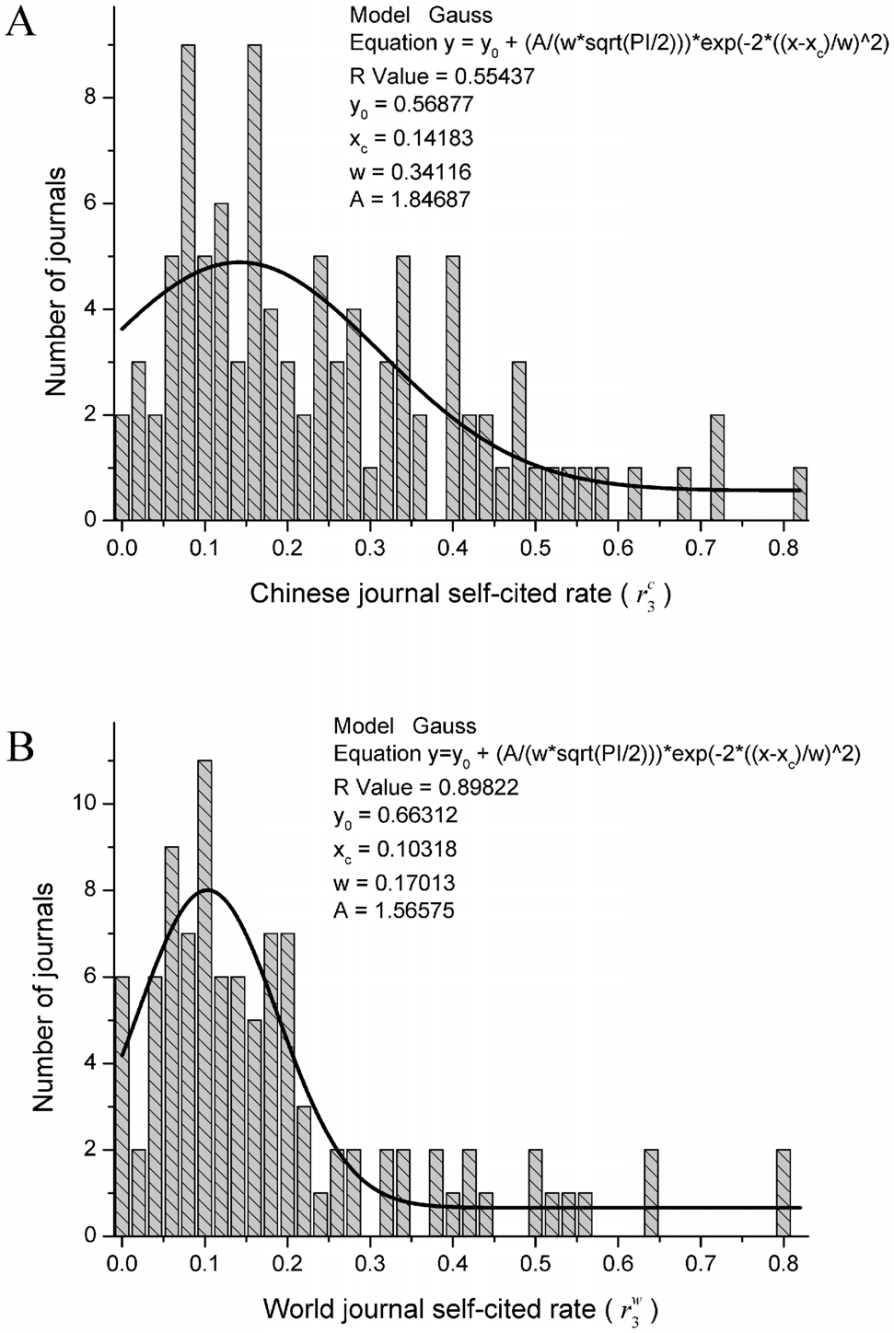
Distributions of the journal self-cited rates (*r_3_*). A) Journal self-citing rates for Chinese journals and B) for world journals. Note that Fig. 3 A can not be well fitted by a Gaussian model, indicating that there probably exist some non-random factors leading to the non-Gaussian distribution. Furthermore, the Chinese journal self-cited rates are generally higher than those of world journals, as reflected by the comparison between the locations of the maximum for both distributions (0.14 versus 0.1).

Fig. 2 shows the distributions of journal self-citing rates for both Chinese and world journals. Similar to Fig. 1 A and B, the distributions shown in Fig. 2 A and B can also be well fitted by a Gaussian model. Similarly, the maximum of the former is still slightly greater than that of the latter. As we can see from Fig. 2 A and B, the maximum of the distribution for Chinese journals is at about 0.008, whereas that for world journals is at about 0.006. Additionally, there is no world journal whose 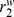 value is greater than 0.04, whereas there are seven Chinese journals whose 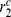 values are greater than 0.04. One Chinese journal even has the journal self-citing rate 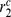 nearly 0.119. Both results indicate that the Chinese journal self-citing rates are generally higher than that of world journals.

Fig. 3 shows the distributions of journal self-cited rates for both Chinese and world journals. It seems that Fig. 3 A can not be well fitted by a Gaussian model. At least, the Gaussian fitting model for Fig. 3 A works worse than that for world journals, indicating that there probably exist some non-random factors leading to the non-Gaussian distribution. Furthermore, the Chinese journal self-cited rates are generally higher than that of world journals, as reflected by the fact that the location of the maximum for the former is greater than that for the latter (0.14 versus 0.1).

**Figure 4:**
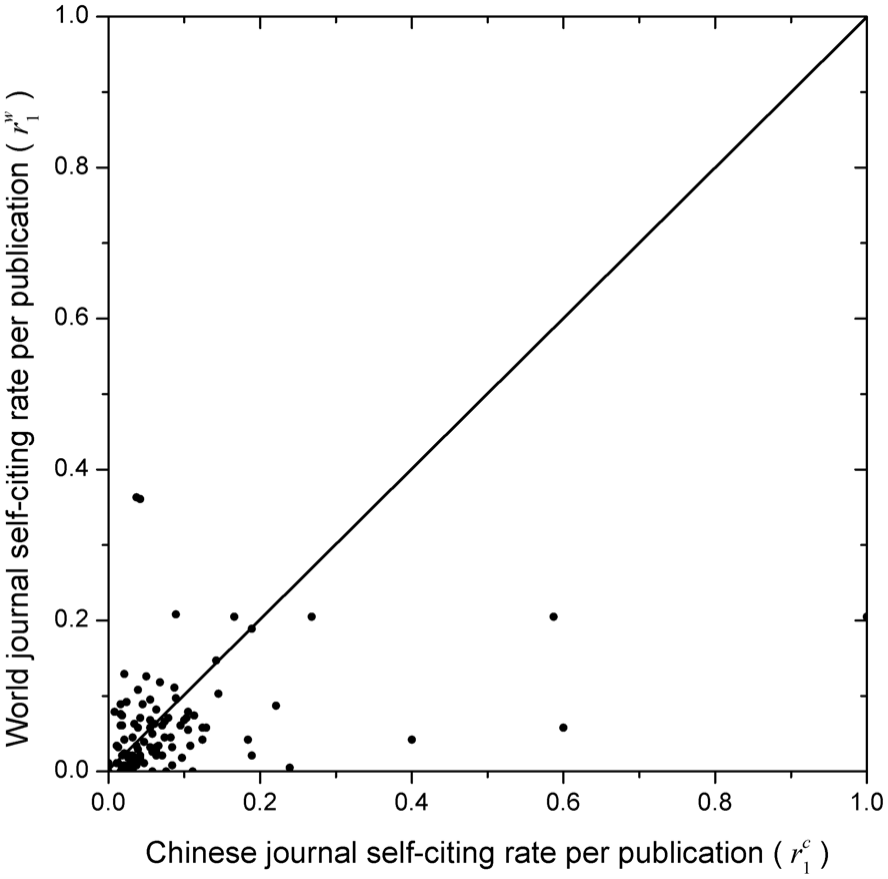
Comparison of the magnitude of the journal self-citing rates per publication. The comparison is between Chinese (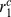) and world journals (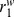), corresponding to the X-axis and Y-axis, respectively. Each Chinese journal and its corresponding world journal constitute a pair of journals, and (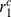,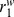) is corresponding to a point in the X-Y plane. Note that there are 62 and 34 points situated at the down (X>Y) and up (Y>X) triangle, respectively, and there are 3 points situated at the diagonal (X = Y). That is to say, for most Chinese journals the journal self-citing rates per publication are higher than those of world journals.

**Figure 5:**
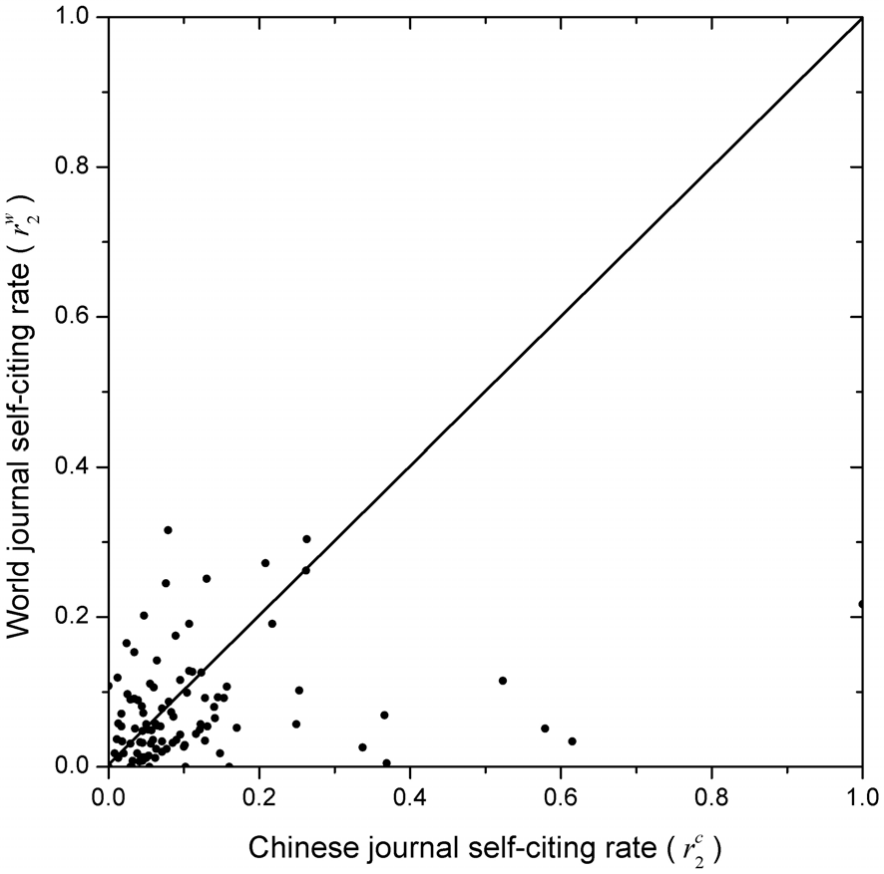
Comparison of the magnitude of the journal self-citing rates. The comparison is between Chinese (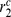) and world journals (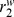), corresponding to the X-axis and Y-axis, respectively. Note that there are 60 and 37 points situated at the down (X>Y) and up (Y>X) triangle, respectively, and there are 2 points situated at the diagonal (X = Y). That is to say, for most Chinese journals the journal self-citing rates are higher than those of world journals.

**Figure 6:**
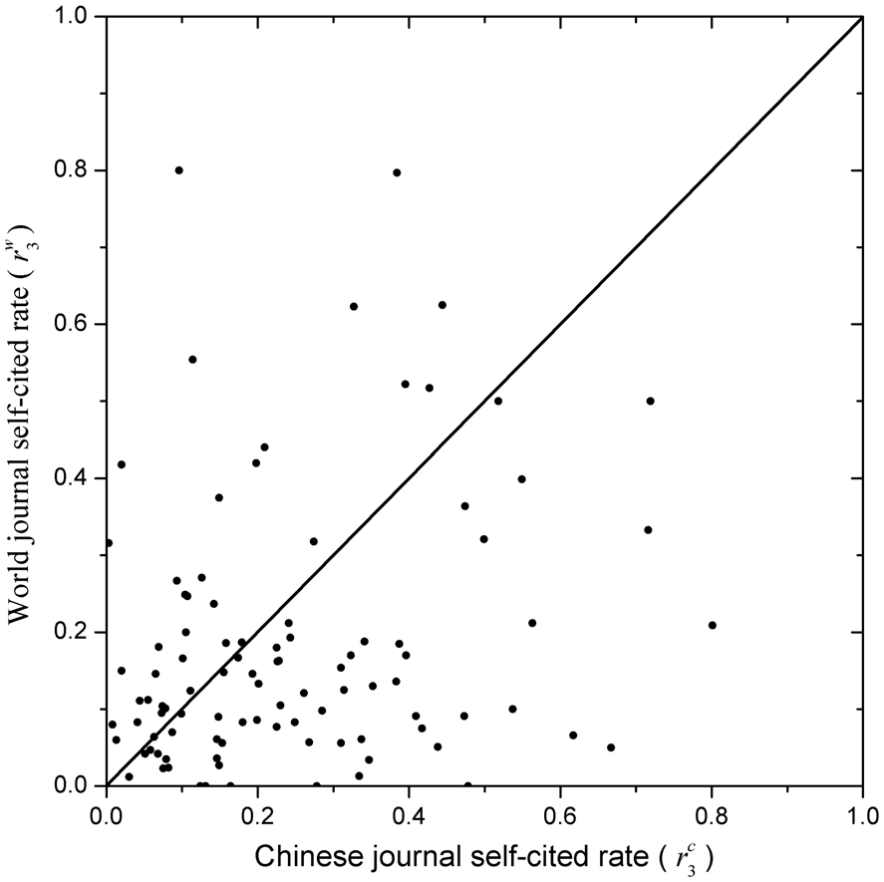
Comparison of the magnitude of the journal self-cited rates. The comparison is between Chinese (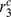) and world journals (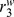), corresponding to the X-axis and Y-axis, respectively. Note that there are 63 and 35 points situated at the down (X>Y) and up (Y>X) triangle, respectively, and there is 1 point situated at the diagonal (X = Y). That is to say, for most Chinese journals the journal self-cited rates are higher than those of world journals.

### Comparison of the Magnitudes of Journal Self-citation Rates between Chinese and World Journals

To compare the magnitudes of journal self-citation rates between Chinese and world journals, refer to Figs. 4–6, which provide a new viewing angle. The ranges of both X-axis and Y-axis of all these figures are from 0 to 1. Note that the journal self-citing rates per publication, 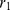, can be greater than 1. For 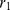 and 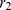, we made a transform as follows
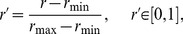
(1)where 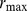 and 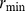 are the maximum and minimum values among the 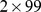 rates. For 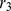, no such transform was made. In each of Figs. 4–6, the square is divided by a diagonal into two triangles, the up and down triangle. The points in the up triangle indicate that Y>X, whereas in the down triangle indicate that X>Y. Referring to Fig. 4 first, the number of points in the down triangle is 62, that in the up triangle is 34, and there are 3 points situated at the diagonal (X = Y). That is to say, for most Chinese journals the journal self-citing rates per publication were higher than those of world journals. Next referring to Fig. 5, the corresponding numbers are 60: 37: 2, indicating that for most Chinese journals the journal self-citing rates are also higher than those of world journals. Finally referring to Fig. 6, it is seen that the corresponding numbers are 63: 35: 1. The same conclusion holds for the journal self-cited rates. In summary, for all three self-citation rates studied in this paper, generally, Chinese journals had higher self-citation rates than their world journal counterparts.

**Figure 7:**
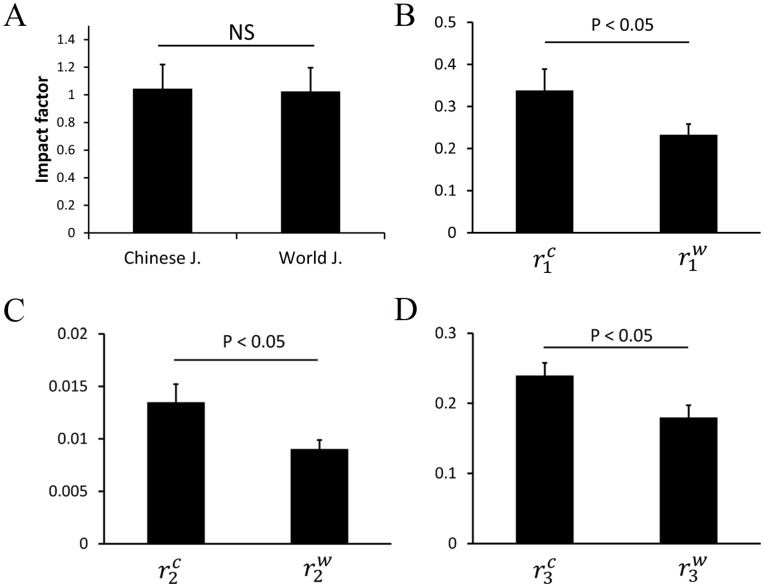
Histogram showing the average values of the three self-citation rates and impact factors for Chinese and world journals. A) Average impact factors for 99 Chinese and 99 world journals are comparable. However, for B) journal self-citing rates per publication (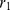), C) the journal self-citing rates (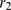) and D) the journal self-cited rates (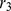), Chinese journals have values greater than those of their world counterparts with p<0.05. Data are represented as mean ± SEM.

**Table 1:** The average and standard deviation of the six rates^a^.

Rate	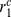	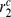	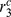	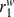	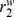	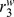
Average value	0.338	0.013	0.240	0.233	0.009	0.180
Standard deviation	0.508	0.017	0.181	0.253	0.008	0.173

a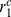 and 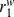 are the journal self-citing rates per publication; 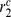 and 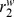 are the journal self-citing rates; and 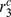 and 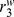 are the journal self-cited rates, for Chinese and world journals, respectively.

The average value and its standard deviation for each of the three rates and for Chinese and world journals, respectively, are listed in Table 1. It can be seen from Table 1 that for each of the three self-citation rates, the average value for Chinese journals was greater than that for their world counterparts, in agreement with the conclusions observed in Fig. 4–6. To test if the results have statistical significance, we performed paired *student’s t*-test, showing that the journal self-citing rates per publication, the journal self-citing rates and the journal self-cited rates of Chinese journals are greater than those of their world journal counterparts (p<0.05) (Fig. 7).

**Table 2:** The correlation coefficients between various journal self-citation rates and impact factor.

Rate	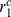	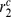	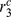	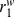	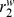	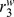
Correlation coefficient	0.216	0.056	−0.223	0.421	0.075	−0.212

**Figure 8:**
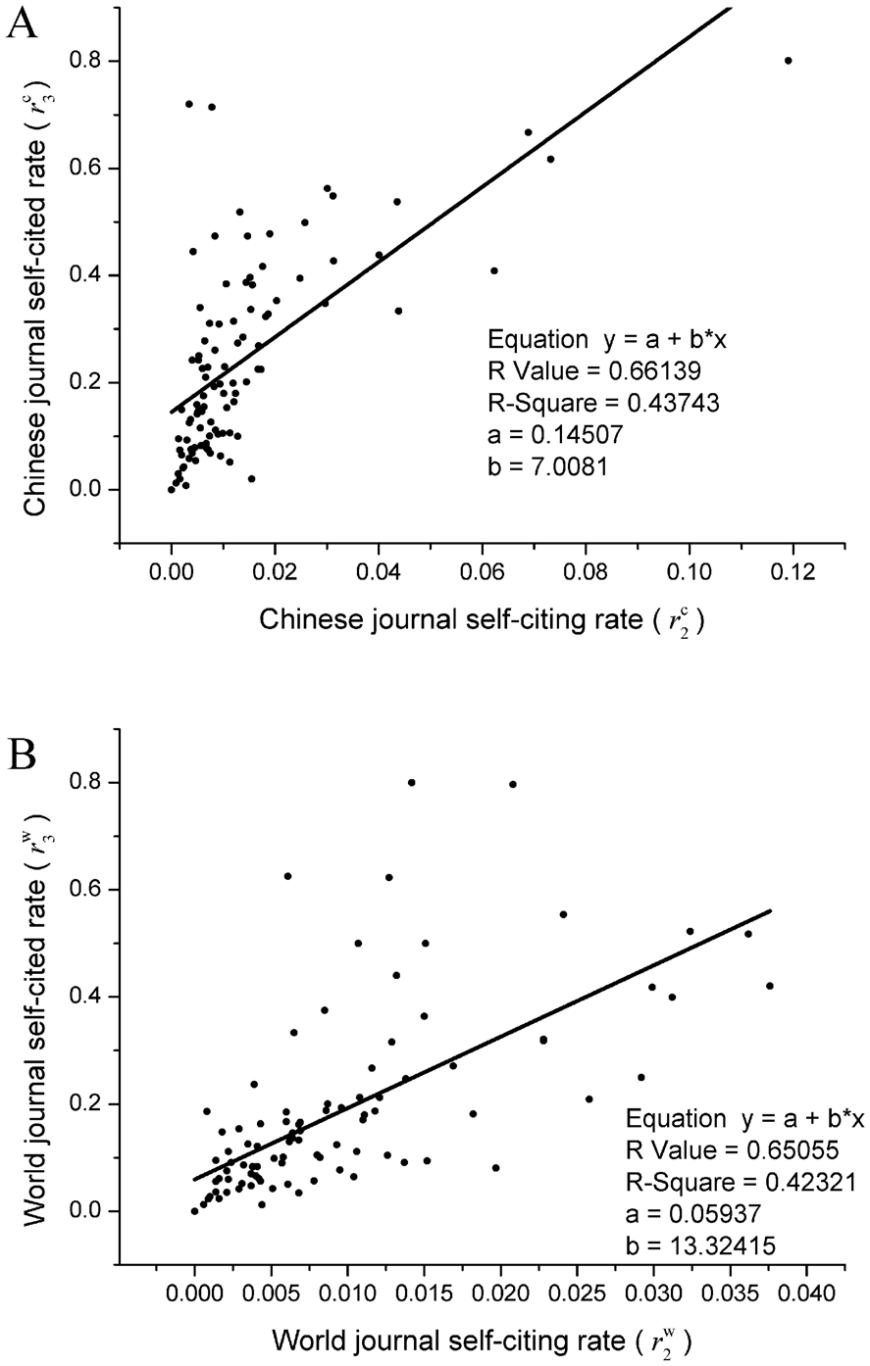
Linear correlation between the journal self-citing rate and self-cited rate for Chinese and world scientific journals. A) For 99 Chinese scientific journals indexed by the JCR, and B) for 99 world scientific journals also indexed by the JCR, there is a linear correlation between the journal self-citing rate and self-cited rate.

### The Relation between Journal Self-citation Rates and Impact Factors

The correlation coefficients between each of the three rates with the impact factor of the corresponding journals are listed in Table 2. It is seen that there existed positive correlation between the journal self-citing rate per publication and impact factor for both Chinese and world journals, with the corresponding correlation coefficients 0.216 and 0.421, respectively. However, there is almost no correlation between the journal self-citing rate and the impact factor for both Chinese and world journals, with the correlation coefficients 0.056 and 0.075, respectively. The most remarkable feature in Table 2 is that the journal self-cited rate is negatively correlated with the impact factor for both Chinese and world journals, with the correlation coefficients −0.223 and −0.212, respectively. This finding is consistent with the result of Biglu [9], who found that the self-cited rate has a negative correlation with impact factor. Biglu hence pointed out that the journals with lower impact factor tend to be cited more by themselves [9]. In what follows, we shall give an explanation of the negative correlation coefficients. Based on the definitions of journal self-citation rates in eqs. (3) and (5), and assuming that the numbers of publications in each of 2008, 2009 and 2010 for a journal are roughly equal, we find



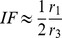
(2)where IF is the journal impact factor. For fixing 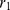, the impact factor IF is reversely proportional to the journal self-cited rate 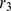. It seems that this formula explains the negative correlation between IF and 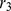.

**Table 3:** List of Chinese and world scientific journals being studied.

No.	Chinese Journals		World Journals	
	JCR Abbrev. Title	IF	JCR Abbrev. Title	IF
1	ACTA BIOCH BIOPH SIN	1.547	BIOMED CHROMATOGR	1.545
2	ACTA CHIM SINICA	0.611	J CHEM EDUC	0.571
3	ACTA MATH APPL SIN-E	0.371	STOCHASTICS	0.369
4	ACTA MATH SCI	0.213	DOKL MATH	0.204
5	ACTA MATH SIN	0.540	PAC J MATH	0.549
6	ACTA MECH SINICA-PRC	0.749	ARCH APPL MECH	0.853
7	ACTA MECH SOLIDA SIN	0.543	J COMPUT NONLIN DYN	0.571
8	ACTA METALL SIN	0.482	PROT MET PHYS CHEM+	0.466
9	ACTA METEOROL SIN	0.704	WEATHER	0.588
10	ACTA OCEANOL SIN	0.476	IZV ATMOS OCEAN PHY+	0.528
11	ACTA PHARMACOL SIN	1.909	J PHARM PHARMACOL	1.918
12	ACTA PHYS SIN-CH ED	1.259	FORTSCHR PHYS	1.144
13	ACTA PHYS-CHIM SIN	0.734	INT J THERMOPHYS	0.750
14	ACTA POLYM SIN	0.481	J POLYM ENG	0.493
15	ADV ATMOS SCI	0.925	PHYS CHEM EARTH	0.917
16	ALGEBR COLLOQ	0.305	MATH SLOVACA	0.316
17	APPL GEOPHYS	0.387	ANN GEOPHYS-ITALY	0.336
18	APPL MATH MECH-ENGL	0.517	INT J NUMER METHOD H	0.527
19	APPL MATH SER B	0.144	MATH COMMUN	0.176
20	ASIAN J ANDROL	1.549	INT UROL NEPHROL	1.567
21	ASIAN PAC J TROP MED	0.172	J VENOM ANIM TOXINS	0.302
22	CELL MOL IMMUNOL	2.026	J INFLAMM-LOND	2.017
23	CELL RES	9.417	PLANT CELL	9.396
24	CHEM J CHINESE U	0.656	RUSS CHEM B+	0.629
25	CHEM RES CHINESE U	0.460	J CHIL CHEM SOC	0.532
26	CHIN J INTEGR MED	0.578	AFR J TRADIT COMPLEM	0.457
27	CHIN J MECH ENG-EN	0.194	MECH ENG	0.250
28	CHIN J OCEANOL LIMN	0.325	OCEANOLOGY+	0.324
29	CHIN OPT LETT	0.694	OPT ENG	0.822
30	CHINA COMMUN	0.058	MICROWAVES RF	0.073
31	CHINA FOUNDRY	0.204	MINER METALL PROC	0.167
32	CHINA OCEAN ENG	0.302	P I CIVIL ENG-MAR EN	0.333
33	CHINA PET PROCESS PE	0.088	CHEM TECH FUELS OIL+	0.053
34	CHINESE ANN MATH B	0.452	PURE APPL MATH Q	0.462
35	CHINESE CHEM LETT	0.775	KOREAN J CHEM ENG	0.748
36	CHINESE GEOGR SCI	0.656	ENVIRON EARTH SCI	0.678
37	CHINESE J AERONAUT	0.301	AERONAUT J	0.496
38	CHINESE J ANAL CHEM	0.798	ANAL LETT	0.920
39	CHINESE J CANCER RES	0.252	ASIA-PAC J CLIN ONCO	0.296
40	CHINESE J CATAL	0.752	INT J THERMOPHYS	0.750
41	CHINESE J CHEM ENG	0.901	CHEM ENG COMMUN	0.913
42	CHINESE J CHEM PHYS	0.642	FULLER NANOTUB CAR N	0.631
43	CHINESE J GEOPHYS-CH	0.832	J GEOPHYS ENG	0.805
44	CHINESE J INORG CHEM	0.670	J RADIOANAL NUCL CH	0.777
45	CHINESE J ORG CHEM	0.555	INDIAN J CHEM B	0.562
46	CHINESE J POLYM SCI	0.478	INT J POLYM MATER	0.458
47	CHINESE J STRUC CHEM	0.624	CRYSTALLOGR REP+	0.644
48	CHINESE MED J-PEKING	0.983	ISR MED ASSOC J	0.953
49	CHINESE PHYS LETT	1.078	PROG THEOR PHYS SUPP	1.017
50	CHINESE SCI BULL	1.087	SCI ENG ETHICS	1.119
51	COMMUN THEOR PHYS	0.488	J KOREAN PHYS SOC	0.478
52	EARTHQ ENG ENG VIB	0.880	J EARTHQ ENG	0.843
53	EPISODES	2.041	GEOARABIA	2.026
54	FRONT MATH CHINA	0.494	IZV MATH+	0.494
55	FRONT PHYS CHINA	0.581	PHYS WORLD	0.561
56	FUNGAL DIVERS	5.074	FUNGAL GENET BIOL	3.333
57	HEPATOB PANCREAT DIS	1.514	HPB	1.285
58	INT J SEDIMENT RES	1.708	ENVIRON GEOCHEM HLTH	1.667
59	J BIONIC ENG	1.032	BIO-MED MATER ENG	1.026
60	J CENT SOUTH UNIV T	0.331	INT J POWDER METALL	0.302
61	J COMPUT MATH	0.760	NODEA-NONLINEAR DIFF	0.770
62	J COMPUT SCI TECH-CH	0.656	J CIRCUIT SYST COMP	0.215
63	J ENVIRON SCI-CHINA	1.513	J ARID ENVIRON	1.535
64	J GENET GENOMICS	1.494	PESTIC BIOCHEM PHYS	1.503
65	J GEOGR SCI	0.673	PHYS GEOGR	0.683
66	J HUAZHONG U SCI-MED	0.405	J PLANT BIOCHEM BIOT	0.412
67	J HYDRODYN	1.475	J FLUID STRUCT	1.482
68	J INFRARED MILLIM W	0.452	LASER FOCUS WORLD	0.353
69	J INORG MATER	0.399	J CERAM PROCESS RES	0.484
70	J IRON STEEL RES INT	0.140	T INDIAN I METALS	0.160
71	J MATER SCI TECHNOL	0.759	MATER TRANS	0.787
72	J MOL CELL BIOL	13.400	CURR OPIN CELL BIOL	13.540
73	J MT SCI-ENGL	0.632	NAT RESOUR MODEL	0.596
74	J NAT GAS CHEM	1.345	INT J PHOTOENERGY	1.345
75	J RARE EARTH	1.086	J COAT TECHNOL RES	1.056
76	J SYST ENG ELECTRON	0.214	IMA J MATH CONTROL I	0.213
77	J SYST SCI COMPLEX	0.564	J MATH ECON	0.549
78	J THERM SCI	0.212	J THERM SCI TECH-JPN	0.250
79	J TROP METEOROL	0.380	RUSS METEOROL HYDRO+	0.232
80	J WUHAN UNIV TECHNOL	0.386	MATER SCI-MEDZG	0.409
81	J ZHEJIANG UNIV-SC A	0.326	COMPUT APPL ENG EDUC	0.321
82	J ZHEJIANG UNIV-SC B	1.027	GENET MOL RES	1.013
83	LIFE SCI J	0.158	ASIA LIFE SCI	0.189
84	MOL PLANT	4.296	PLANT CELL PHYSIOL	4.257
85	NANO RES	5.078	NANOMED-NANOTECHNOL	4.882
86	NEURAL REGEN RES	0.180	NEUROCHEM J+	0.151
87	NEW CARBON MATER	0.888	FATIGUE FRACT ENG M	0.894
88	NUCL SCI TECH	0.204	ATOM ENERGY+	0.071
89	PARTICUOLOGY	1.317	INT J NANOTECHNOL	1.335
90	PEDOSPHERE	0.978	SOIL SCI	0.923
91	PETROL SCI	0.432	SPE PROD OPER	0.360
92	PLASMA SCI TECHNOL	0.553	PLASMA PHYS REP+	0.668
93	PROG BIOCHEM BIOPHYS	0.236	DOKL BIOCHEM BIOPHYS	0.331
94	PROG CHEM	0.560	J CHEM RES	0.550
95	RARE METAL MAT ENG	0.139	METAL INT	0.154
96	RARE METALS	0.643	J MATER ENG PERFORM	0.639
97	T NONFERR METAL SOC	0.677	MATER SCI TECH-LOND	0.709
98	WORLD J GASTROENTERO	2.240	J GASTROEN HEPATOL	2.410
99	WORLD J PEDIATR	0.945	ANN TROP PAEDIATR	0.966

### The Relation between Journal Self-citing Rates and Self-cited Rates

As mentioned above, it was found that there is a linear correlation between journal self-citing and self-cited rates [8]. Perhaps, this is a basic characteristic of the self-citation of journals. The present study provides an excellent opportunity to study this phenomenon. We also found that there is a linear correlation between self-citing and self-cited rates for both 99 Chinese and 99 world scientific journals. Refer to Fig. 8. It can be seen that in both cases, the data are well fitted by a straight line, with 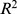 = 0.437 and 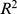 = 0.423, respectively, for the 99 Chinese and world scientific journals. Based on the above results, we conclude that the linear correlation between self-citing rate and self-cited rate of journals is a basic characteristic of journal self-citations.

### Concluding Remarks

The three journal self-citation rates are comprehensively compared between Chinese and world scientific journals. Note that the 99 Chinese journals almost exhausted all Chinese journals indexed by the 2010 JCR, and therefore they were appropriate representatives of Chinese scientific journals then available. Generally speaking, most Chinese scientific journals have higher self-citation rates than their world counterparts. According to Rousseau [4], higher self-citing rate indicates more isolation in the relevant field covered by the journal, and higher self-cited rate indicates a journal’s lower visibility. Our results suggest that Chinese scientific journals generally have lower visibility than their world journal counterparts, and are more isolated in the relevant fields. We emphasize that the study results presented in this paper are limited in that they refer to the particular year 2010, citing the papers published in 2008 and 2009. Therefore, caution needs to be taken not to over-interpret the results.

The low visibility and severe isolation are caused by many reasons, which can include the followings. First, editorial boards of Chinese journals are usually dominated by Chinese researchers, leading to relative isolation from international peers. Second, it is not uncommon for manuscripts written by Chinese authors to have English usage problems, lowering their readability. Third, Chinese journals are commonly distributed within China, leading to low visibility from international scientific community. Forth, for some Chinese journals not adopting an open access policy, the payment process can be inconvenient for international users.

Accordingly, here we would like to put forward some suggestions, which hopefully are helpful to solve these problems. First, we suggest journal editors to invite international experts to join the editorial board, in addition to having Chinese experts. Second, journals may implement a policy of using mandatory language polishing services before publishing manuscripts. We further suggest that some Chinese scientific agencies may consider providing funding mechanisms to help journals provide language polishing services for free. Third, it would be helpful for journals to seek international agencies, professional societies and prominent publishers to promote and distribute the journals to a broader audience. Forth, we suggest that Chinese funding agencies can consider adopting an open access policy, similar to the one being used by the National Institute of Health (NIH) in the United States. To ensure public access of published results from NIH funded research, NIH requires scientists to deposit accepted manuscripts to PubMed Central. If Chinese funding agencies adopt this public access policy, it not only helps to advance science, but also helps to broaden the visibility of Chinese scientific journals.

Considering the fact that sciences are rapidly developing in China and so are Chinese scientific journals, we hope and believe that these problems will be gradually solved. It is expected that the differences of journal self-citation rates between Chinese and world scientific journals will gradually disappear in the future.

## Materials and Methods

### Definition of Journal Self-citation Rates

This paper is devoted to studying the journal self-citation rates, which, similar to the author self-citations, also belong to a subject of citation analysis [4]. Note that the journal self-citation rates are divided into two categories: self-citing rates and self-cited rates [4]. It is obvious that both rates are real numbers between 0 and 1. Since the number of citations is related to the time window used for the calculation, the details need to be specified. In what follows, we will focus on the self-citation rates in the year 2010. To be consistent with the definition of the impact factor in 2010, for a given journal we study the citations in the year 2010 to the papers published in both 2008 and 2009 in the journal under study. A total of three journal self-citation rates are studied in this paper. Their definitions are detailed as follows.

1)The journal self-citing rate per publication, denoted by 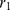,
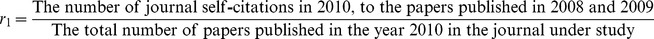
(3)


2) The journal self-citing rate, denoted by 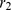,
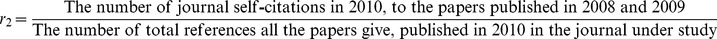
(4)


3) The journal self-cited rate, denoted by 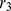,
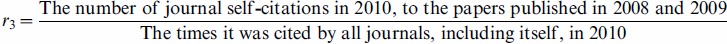
(5)


Note that 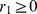, whereas 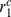. Hereafter we will neglect the detailed year information such as 2008, 2009 and 2010, but it should be emphasized that the journal’s self-citations we study here are those particularly calculated in the year 2010.

### Journals Selected for Study

There were 138 journals listed in “countries/territories PEOPLES R CHINA” in the JCR Science Edition 2010. Among the 138 journals, a few of them changed their journal’s titles during the period of 2008–2010. Some journals were edited and published by Hong Kong institutes, and were excluded from the current study. Consequently, 99 Chinese scientific journals remained and were studied in this paper. The related information of them, including their titles and impact factors, are listed in Table 3. For the purpose of comparison, 99 world or international, non-Chinese journals were also selected. For each Chinese journal, the corresponding world journal was in the same JCR subject category as its Chinese counterpart with similar impact factor. Even with these criteria, there are still a few possible choices. We selected one of the possible world journals randomly. Similarly, the related information of these 99 world journals is also listed in Table 3. One Chinese journal and its corresponding world journal constituted a pair of journals, used for comparison in the present study. For convenience, the journal self-citing rate per publication is denoted by 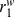 and 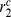; the journal self-citing rate is denoted by 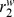
and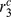; and the journal self-cited rate is denoted by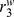 and 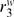, respectively, for Chinese and world journals.

### Calculation Method

All of the data used in this study were collected from JCR Science Edition 2010 in the ISI Web of Knowledge. The details are as follows. “PEOPLES R CHINA” in the country/territories list was selected in the option of “View a group of journals by Country/Territory”. There were 138 journals of PEOPLES R CHINA in the “Journal Summary List”. For each journal, we collected basic information such as “Impact Factor”, “Articles and Reviews Numbers in JCR year 2010” (i.e., the denominator of eq. (3)), and “Number of references” (i.e., the denominator of eq. (4)) from the “Journal Information” page. Then, from the “Cited Journal data table”, we collected the “number of journal self-citations in 2010, citing the papers published in 2008 and 2009” (i.e., the numerators of eqs. (3)–(5)) and the “number of citations citing the papers published in 2008 and 2009 by all journals, including itself, in 2010” (i.e., the denominator of eq. (5)). All the collected data were input into an Excel sheet and then processed. The journal self-citation rates, i.e., the journal self-citing rate per publication, the journal self-citing rate and the journal self-cited rate are defined in eqs. (3), (4) and (5), respectively. The calculations are simple and trivial.
